# The role of dorsal raphe nucleus neuropeptides in reward and aversion

**DOI:** 10.3389/fnbeh.2025.1553470

**Published:** 2025-04-09

**Authors:** Kathryn Braden, Daniel C. Castro

**Affiliations:** Mallinckrodt Institute of Radiology, Washington University School of Medicine, Saint Louis, MO, United States

**Keywords:** neuropeptide, dorsal raphe nucleus, reward, aversion, behavior

## Abstract

The dorsal raphe nucleus is a critical node for affective and motivated circuits in the brain. Though typically known as a serotonergic hub, the dorsal raphe nucleus is also highly enriched in a variety of neuropeptides. Recent advances in biotechnology and behavioral modeling have led to a resurgence in neuropeptide research, allowing investigators to target unique peptide systems with unprecedented clarity. Here, we review and discuss multiple neuropeptide systems in dorsal raphe and consider how their activity may contribute to reward and aversion. While this is not an exhaustive review, this short overview will highlight the many opportunities available to refine our understanding of multiple dorsal raphe neuropeptides. By more thoroughly studying dorsal raphe neuropeptides, we will reveal novel pathways to design more effective therapeutics and tailor treatments for millions of patients.

## 1 Introduction

The dorsal raphe nucleus (DRN) in the midbrain has been implicated in reward processing, mood regulation, and learning (Luo et al., 2015; Luo et al., 2016; McDevitt and Neumaier, 2011). It is one of seven raphe nuclei along the midsagittal plane of the brainstem that are characterized by the presence of serotonin positive (5-hydroxytryptamine; 5-HT) cell bodies (Dahlström and Fuxe, 1964; Taber et al., 1960). Of the raphe nuclei, the DRN provides the largest source of serotonin to the forebrain (Vertes, 1991). As a result, the DRN serotonergic system has been the subject of intense interest, particularly in the realm of affect and motivation. Serotonin was first discovered in 1948 as a vasoconstrictor (Rapport et al., 1948a; Rapport et al., 1948b; Rapport et al., 1948c; Twarog and Page, 1953). Further investigation of serotonergic cells within the raphe found extensive projections throughout the forebrain and cortex, indicating that it could play an important modulatory role in affect, motivation, and learning (Dahlström and Fuxe, 1964). Decades of research more or less corroborate that hypothesis, with the advent of SSRIs and other serotonergic drugs now used regularly in the clinic to treat mood disorders like depression, eating disorders, and anxiety. While clearly a critical node within affective neural circuits, the myopic focus on serotonin has occluded an appreciation for the full neurochemical landscape present within the DRN. Studies find that approximately one to two thirds of DRN neurons are non-serotonergic (Castro et al., 2021; Wiklund et al., 1981), but are neuropeptidergic, leaving unresolved how a significant portion of the dorsal raphe complex may or may not be involved in behavioral phenotypes. For example, despite the presence and high expression of several neuropeptides like the endogenous opioids, cholecystokinin (CCK), and neuropeptide Y (NPY), almost nothing is known regarding the function of these and other neuropeptides in this region.

Neuropeptides are small proteins derived from post-translational processing of precursor proteins that are released from neurons in dense core vesicles to act on receptors, typically G-protein coupled receptors (GPCRs) (Russo, 2017). There are at least 50 neuropeptides expressed in the mammalian brain, each with varying levels of understanding of their physiological roles (Eiden et al., 2022). These signaling molecules can have significant modulatory effects on neuronal transmission and resulting behavioral phenotypes. In many cases, neuropeptides are co-released by neurons along with classical neurotransmitters such as glutamate, GABA, dopamine, or serotonin (Eiden et al., 2022; Nusbaum et al., 2017). Co-release of neuropeptides and neurotransmitters enable highly nuanced circuit flexibility, as peptides can have both neurotransmitter dependent or independent mechanisms of action. Details regarding co-transmission and other peptide-specific signaling mechanisms are reviewed thoroughly elsewhere (Eiden et al., 2022; Nusbaum et al., 2017; Russo, 2017; Svensson et al., 2019), but are critical elements to consider in the context of neuropeptide function in the DRN.

This review aims to highlight the current literature on a selection of neuropeptides within the DRN and describe their known anatomical, physiological, and functional characteristics. Below we outline what is known about the general structure and connections of the DRN, we then summarize the current literature of specific neuropeptides and their functional effects in this region. Overall, we aim to emphasize that DRN neuropeptides act both within and beyond classic serotonergic systems, and provide important contributions to reward, motivation, and affect that could be leveraged in the treatment of neuropsychiatric conditions.

## 2 Structure and connectome of DRN

**FIGURE 1:**
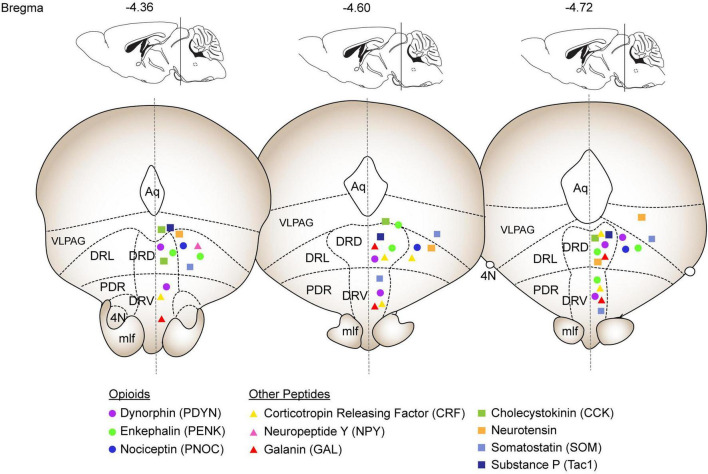
Summary of expression patterns of selected neuropeptides throughout the DRN. The coronal sections and annotations are based on the Franklin & Paxinos mouse brain atlas and depict the DRN from –4.36 to –4.72 Bregma. Opioid peptides are depicted as circles (Dynorphin: purple, Enkephalin: green; Nociceptin: blue). The additional peptides discussed in depth are depicted as triangles (Corticotropin releasing factor: yellow; Neuropeptide Y: pink; Galanin: red). The peptides discussed in the Other Neuropeptides section are depicted as squares (Cholecystokinin: green; Neurotensin: orange; Somatostatin: light blue; Substance P: dark blue). Abbreviations: Aq: Aqueduct, VLPAG: ventrolateral periaqueductal grey, DRL: dorsal raphe lateral, DRD: dorsal raphe dorsal, DRV: dorsal raphe ventral, PDR: postero-dorsal raphe, 4N: trochlear nucleus, mlf: medial longitudinal fasciculus.

The DRN can be divided into medial and lateral regions as well as rostral and caudal portions. It is located directly below the cerebral aqueduct beginning in the midbrain at the caudal border of the oculomotor nuclei and extending to the mid-pons where the aqueduct meets the fourth ventricle (Franklin and Paxinos, 2007; Jacobs and Azmitia, 1992). It is located directly along the midline with the medial portion extending from the aqueduct superiorly to surround the medial longitudinal fasciculi (MLF) inferiorly. The lateral wings form the largest portion of the DRN and extend out to meet the ventrolateral periaqueductal gray (vlPAG) at the level of the trochlear nuclei until just below the fourth ventricle. Importantly the border between the DRN and vlPAG is not consistently defined; early studies investigating the vlPAG included portions of what would now be considered the DRN (Kyuhou and Gemba, 1999; Monassi et al., 1999; Siegel and Pott, 1988; Yaksh et al., 1976). Recent studies have begun to treat the vlPAG and lateral DRN (LDRN) as a complex of cells that work together, perhaps acting as a functionally unique transition zone (Dougalis et al., 2012; Liu et al., 2023; Xie et al., 2023). These sorts of transitions zones have been described in a variety of mesocorticolimbic systems, including caudal accumbens into rostral bed nucleus of the solitary tract or portions of the extended amygdala (Thompson and Swanson, 2010; Zahm et al., 2013). But for the purposes of this review, we will refer to the LDRN as separate from the vlPAG and adhere to the annotations of Franklin and Paxinos to approximate this border (Figure 1; Franklin and Paxinos, 2007).

### 2.1 Connections of the DRN

As would be expected for the primary source of serotonin to the brain, the DRN is highly interconnected with many brain regions with known roles in reward and aversion, including the nucleus accumbens (NAc) (Castro et al., 2021; Pomrenze et al., 2022; Vertes, 1991), ventral tegmental area (VTA) (Abraham et al., 2022), amygdala (Marcinkiewcz et al., 2016; Ren et al., 2018; Ritchie et al., 2024), and PAG (Xu et al., 2021). The projections are generally topographically organized, with more rostral DRN neurons projecting to cortical and subcortical regions and caudal neurons projecting to septohippocampal structures (Commons, 2020). The nonserotonergic neurons have not been specifically characterized for potential functional subgroups yet (Huang et al., 2019). Most tracing studies in the literature have focused on serotonergic cells, but recent work has made efforts to more thoroughly characterize the inputs and outputs of the entire DRN by comparing glutamatergic and GABAergic cell types (Xu et al., 2021). This in-depth mapping of whole-brain connections of the DRN highlights the complexity of interconnectedness present in this highly heterogenous nucleus. For more thorough review of the current understanding of dorsal raphe circuitry we suggest the recent reviews by Steinbusch and colleagues (Steinbusch et al., 2021) or Zhang and colleagues (Zhang et al., 2024a).

### 2.2 Cellular characterization of DRN neurons

As early as the studies of Cajal, the neurons of the DRN as it is known today were grouped into three to four morphological categories: fusiform, multipolar (stellate), and ovoid or triangular cells (Andrade and Haj-Dahmane, 2013; Baker et al., 1990; Michelsen et al., 2007). These neurons have divergent and spiny dendrites with fibers concentrating in either ascending or descending bundles. To date, most efforts into the anatomical classification of dorsal raphe neurons have focused on subtyping serotonin cells, the majority of which are clustered densely along the midline and are present throughout the lateral wings (Andrade and Haj-Dahmane, 2013). Though not well described, one study found that nonserotonergic neurons from the DRN had fewer dendritic spines than serotonin-positive cells. Additionally, nonserotonergic neurons had more heterogenous electrophysiological properties than the slow rhythmic firing that is often used to characterize serotonergic cells (Li et al., 2001). However, more recent studies have found that even serotonin neuron firing patterns may be more heterogenous than traditionally thought (Calizo et al., 2011; Schweimer et al., 2011). These studies underscore the importance of parsing how 5-HT and non-5-HT cells in the DRN can be functionally distinguished from each other.

The advent of single cell sequencing has recently led to several studies attempting a more sophisticated characterization of neuronal subtypes within the DRN. But unfortunately, the focus has once again proven limited with the majority using chemical or genetic manipulations to select for only serotonergic cells in their downstream analyses of neurotransmitter expression (Okaty et al., 2020; Ren et al., 2019), rather than considering other neuronal cell types that are present in the DRN. While there is an active and evolving conversation attempting to define and categorize DRN neurons, consensus has not yet been reached (Andrade and Haj-Dahmane, 2013; Commons, 2020; Huang et al., 2019; Monti, 2010). Whether they are divided by transcriptional profile, projection pattern, or anatomical location, there appears to be a marked heterogeneity to these neurons suggesting complex and integrative functional roles that cannot be simplified to 5-HT expression alone.

## 3 Neuropeptides within the DRN

Many studies have found expression of neuropeptides such as neuropeptide Y (NPY), Substance P, dynorphin, enkephalin, and corticotropin releasing factor expressed throughout the DRN (Commons et al., 2003; de Quidt and Emson, 1986; Fu et al., 2010; Hökfelt et al., 1977; Hökfelt et al., 1978; Uhl et al., 1979a). However, little effort has been made to distinguish their functions apart from the serotonergic neurons in this region. Recent evidence points to neuropeptides within the DRN having powerful effects on motivation independent of serotonin effects (Castro et al., 2021; Li et al., 2016; Nectow et al., 2017). Here we have summarized what has been revealed thus far about neuropeptide expression and function within the DRN and emphasize the importance of understanding how nonserotonergic signals act in concert and in parallel to traditional serotonergic systems. Due to the lack of specific studies, we have focused on four peptide families with more substantial literature on their role in the DRN and included a final section of “Other Neuropeptides” describing peptides with less extensive investigation into their roles in DRN circuitry. Due to the lack of quantitative data on many of these peptides in the DRN, we have summarized their general expression patterns of their mRNA throughout the DRN in Figure 1 based on Allen Brain Atlas in situ data (Lein et al., 2007).

### 3.1 Endogenous opioids

#### 3.1.1 Expression patterns

The endogenous opioid system is comprised of the four precursor peptides proopiomelanocortin (POMC), preproenkephalin (Penk) preprodynorphin (Pdyn), and prepronociceptin (Pnoc). Correspondingly, there are four Gi-coupled receptors, mu opioid receptor (MOPR), delta opioid receptor (DOPR), kappa opioid receptor (KOPR), and nociceptin opioid receptor (NOPR). Decades of research show that opioids can powerfully influence affective and motivated behaviors via their actions in cortex (Castro and Berridge, 2017; Cole et al., 2024; Mena et al., 2013), striatum (Al-Hasani et al., 2015; Bakshi and Kelley, 1993; Castro and Berridge, 2014; Castro et al., 2021; Guy et al., 2011; Massaly et al., 2019; Ragnauth et al., 2000; Toddes et al., 2021), amygdala (Lichtenberg and Wassum, 2017; Nygard et al., 2016), ventral pallidum (Smith and Berridge, 2007; Smith et al., 2009; Wulff et al., 2019), ventral tegmental area (Bals-Kubik et al., 1993; Echo et al., 2002; Margolis et al., 2014; Thomas et al., 2022), and periaqueductal gray (Brandão, 1993; Sante et al., 2000). Like these mesocorticolimbic regions, dorsal raphe is highly enriched in opioids. Both enkephalin and nociceptin appear to be primarily expressed in the lateral wings, extending into vlPAG (Castro et al., 2021). In contrast, dynorphin is exclusively expressed along the midline (Figure 1) and appears to partially colocalize with serotonergic neurons (Pomrenze et al., 2022). Receptor mRNA for MOPR, KOPR and NOPR has been found throughout dorsal raphe, with NOPR additionally having strong expression along the midline (Mansour et al., 1994a; Neal et al., 1999). Surprisingly, DOPRs do not appear to be endemic to dorsal raphe, although that does not preclude potential DOPR expression on incoming terminals. Oppositely, while MOPR mRNA is expressed in a large proportion of dorsal raphe neurons (about 30%) (Castro et al., 2021), receptor binding is fairly low (Mansour et al., 1994b). This could indicate that MOPR proteins may be enriched on downstream terminals rather than cell bodies. These MOPR-expressing DRN neurons have been found to be largely non-overlapping with serotonergic markers, but rather distributed between glutamatergic and GABAergic cells (Castro et al., 2021; Welsch et al., 2023b). Regardless, ample evidence indicates that the dorsal raphe nucleus is enriched in endogenous opioids, suggesting a potential role for this system in regulating behavioral phenotypes.

#### 3.1.2 Functional effects

Dorsal midbrain opioids have been known to modulate behavior for quite some time, but most of these effects have been attributed to the periaqueductal gray (PAG), with the most well-characterized function being the descending modulation of nociception. However, a reevaluation of the literature from the 1980s and 1990s indicates that papers that ostensibly targeted the ventrolateral PAG very likely hit portions of the lateral dorsal raphe (Fardin et al., 1984; Monassi et al., 1999; Sim and Joseph, 1991; Smith et al., 1994). Here, opioid agonists were generally shown to suppress evoked behaviors, such as suppressing electrically stimulated defensive behaviors in cats, vocalizations in guinea pigs, or food intake in rats (Jenck et al., 1987; Kyuhou and Gemba, 1999; Siegel and Pott, 1988). More recent studies using specific targeting have focused mostly on the opioid receptors rather than the peptides. In general, MOPR activation in the DRN appears to potentiate reward behaviors, and produce antinociception (Castro et al., 2021; Ferreira and Menescal-de-Oliveira, 2014; Welsch et al., 2023b). Negative states such as withdrawal and food deprivation may impair the MOPR-expressing neurons in the DRN (Welsch et al., 2023a). Additionally morphine withdrawal studies have indicated that opioid receptors interact with the DRN serotonergic system to modulate behavioral outputs as well as neuronal function (Jolas and Aghajanian, 1997; Li et al., 2024; Lunden and Kirby, 2013; Welsch et al., 2023a). Similar to its role in other regions of the reward system, KOPR agonism in the DRN has been characterized as an opponent process relative to MOPR. For example, whereas MOPR stimulation increases serotonin release, KOPR stimulation suppresses it (Tao and Auerbach, 2005; Tao et al., 2007). NOPR DRN effects have yet to be fully explored, with just a few studies indicating that it is modulated during state changes and antagonism may be protective against antinociceptive tolerance to morphine (Ge et al., 2007; Le Maître et al., 2013; Przydzial et al., 2010).

Unlike opioid receptors, which have been readily targetable with selective pharmacology for decades, opioid peptides have been far more difficult to study. Enkephalin in particular poses difficulty as there are several splice variants that can have different receptor affinities that can have profound effects on functional interpretability. However, recent developments in opioid peptide technology, such as the development of preproenkephalin-cre (Penk-cre) mice and CRISPR-Cas9 knockdown viral vectors, have allowed for the discovery of unique circuit mechanisms important for motivated behaviors. For example, dorsal raphe enkephalinergic projections to the NAc have been found to modulate reward consumption (Castro et al., 2021). Parallel opioid circuit mechanisms have also been described. For example, infusion of nociceptin into the DRN decreases 5-HT neuron activity and downstream serotonin release in the NAc (Nazzaro et al., 2009; Tao et al., 2007). Similarly, a population of dynorphin expressing DRN neurons were found to modulate opioid withdrawal-induced social deficits via KOPR-mediated serotonin release to the NAc (Land et al., 2009). Dynorphin, and its associated KOPR, has been additionally implicated in regulating stress responses, as stress manipulations and CRF injections into the DRN caused dynorphin-dependent KOR activation (Land et al., 2008). Other populations of DRN dynorphin neurons projecting to the VTA activate KOPR to modulate dopamine-dependent enhancement of drug reward learning (Abraham et al., 2022). Ultimately, while several local and circuited-based opioidergic mechanisms have been uncovered over the years, there remains extensive gaps in knowledge on their precise cellular mechanisms. Moving forward, it is imperative that we continue to isolate these mechanisms, as well as integrate their function with known roles for other complementary systems, such as the serotonin and GABA/glutamate.

### 3.2 Corticotropin releasing factor

#### 3.2.1 Expression patterns

Corticotropin releasing factor (CRF) has long been studied in the context of stress and motivation (Hupalo et al., 2019; Valentino et al., 2010). First identified in dorsal raphe in 1984 (Merchenthaler, 1984), the peptide and its receptors (CRF1 and CRF2) have been extensively characterized in multiple vertebrates (Alderman and Bernier, 2007; Chappell et al., 1986; Lim et al., 2005; Mancera et al., 1991; Palkovits et al., 1985; Shu et al., 2015). The expression patterns in the DRN appear to be state-dependent, with stress or aversive states modulating expression and localization of both the CRF peptide and its receptors (Chappell et al., 1986; Waselus et al., 2009). In general, CRF precursor mRNA has been found in the medial DRN. In contrast, CRF receptors have a more complex expression pattern that is dependent on the receptor subtype. Specifically, CRF1 is sparsely present and partially colocalized with 5-HT neurons, whereas CRF2 mostly colocalizes with 5-HT in rostral DRN and colocalizes with GABAergic cells in caudal regions (Day et al., 2004).

#### 3.2.2 Functional effects

Early research indicated that DRN CRF was highly sensitive to stressors, potentially indicating that it may interact with serotonin systems to modulate reward or aversive processing. Support for this hypothesis gained traction in the 1990s and 2000s, wherein CRF stimulation was shown to modulate serotonin output and neural activity (Calizo et al., 2011; Kirby et al., 2000; Kovács et al., 2025; Lowry et al., 2000; Lukkes et al., 2009b; Pernar et al., 2004; Price et al., 1998; Thomas et al., 2003). Further work revealed that CRF primarily impacted serotonin activity by increasing GABA release (CRF1 mediated) and sensitivity (CRF1 and CRF2 mediated), as well as effecting other surrounding cell types (Kirby et al., 2008). Functionally, strong associations between generalized stress responses and DRN CRF systems have been well described (Marcinkiewcz et al., 2016). For example, restraint and forced swim stress models elevate dorsal raphe c-fos expression in a CRF dependent manner (Roche et al., 2003; Summers et al., 2018). Maternal aggression and novel object exploration is reduced after CRF administration, and CRF alone is sufficient to generate a conditioned place avoidance. These results potentially point toward CRF increasing anxiety and goal directed avoidance (Clark et al., 2007; Gammie et al., 2004; Land et al., 2008; Wood et al., 2013). Correspondingly, CRF antagonism prevents behavioral deficits due to strong stressors, such as normally increased defensive behaviors in socially defeated hamsters (Cooper and Huhman, 2007) or social deficits after early-life social isolation (Bledsoe et al., 2011; Lukkes et al., 2009a). These and other studies implicating CRF as an important modulator of stress resilience in other brain regions (Chudoba and Dabrowska, 2023) warrant further study of how the DRN CRF system promotes stress coping behaviors. Beyond endogenous or social stressors, DRN CRF also appears to modulate responses to various drugs of abuse, particularly in the context of stress. This appears to hold true for a variety of drugs, including alcohol (Hwa et al., 2013; Knapp et al., 2011; Quadros et al., 2014), psychostimulants (Reinbold Scholl et al., 2014; Verheij et al., 2018; Zorrilla et al., 2012), and opioids (Lunden and Kirby, 2013; Ritchie et al., 2024; Staub et al., 2012). Future work incorporating polysubstance models would be particularly interesting, as this may reveal unexpected synergistic or antagonistic phenotypes.

Though beyond the scope of this review, it is notable that there appears to be regional and anatomical heterogeneity of CRF effects across the DRN (Day et al., 2004; Li et al., 2021; Salvatore et al., 2018; Valentino et al., 2001; Waselus et al., 2005; Waselus et al., 2009). Such functional localization has been observed with other neuropeptides (e.g., opioids) in several other brain regions (Al-Hasani et al., 2015; Castro and Berridge, 2014; Castro and Berridge, 2017; Castro et al., 2016; Parker et al., 2019; Peciña and Berridge, 2005; Smith and Berridge, 2005), but whether these effects map on to DRN-mediated behavioral phenotypes remains to be tested.

### 3.3 Galanin

#### 3.3.1 Expression patterns

Galanin is an evolutionarily conserved peptide that is typically associated with areas like the hypothalamus and locus coeruleus. However, multiple neuroanatomical mapping studies across mammalian and non-mammalian vertebrates show significant galanin peptide and receptor expression in DRN (Figure 1; Araneda et al., 1999; Gundlach et al., 1990; Holmqvist and Carlberg, 1992; Lu et al., 2005b; Melander et al., 1986; Mennicken et al., 2002; Skofitsch and Jacobowitz, 1985; Smith et al., 1994; Sutin and Jacobowitz, 1988). In DRN, galanin primarily colocalizes with serotonergic neurons (Xu and Hökfelt, 1997). However, there is also significant innervation of galanin in DRN that appears to be separate from DRN-derived galanin, indicating that there may be multiple galanin subsystems associated with this region (Xu et al., 1998). Support for this idea can be observed with exogenous galanin infusion in DRN. Here, galanin inhibits 5HT+ neurons and decreases the expression of 5HT1A receptor and galanin mRNA in cell bodies in a GalR3, but not GalR1 dependent manner (Kehr et al., 2002; Razani et al., 2000; Sharkey et al., 2008; Xu et al., 1998). These effects are thought to arise via differential expression of the receptors on presynaptic (GalR1) versus postsynaptic (GalR2/3) membranes (Swanson et al., 2005). However, it should be appreciated that this system is likely highly nuanced, as GalR2 agonism and GalR1/GalR3 antagonism both reduce anxiety and depressive-like phenotypes (de Souza et al., 2018; Lu et al., 2005a; Morais et al., 2016; Silote et al., 2013).

#### 3.3.2 Functional effects

Regardless of the precise mechanism, galanin administration in DRN generally appears to ameliorate aversive phenotypes (García-Durán et al., 2021). Notably, galanin and galanin receptor expression is dynamic and responsive to stress and therapeutic interventions. For example, painful capsaicin exposure increases DRN galanin (Okere and Waterhouse, 2006), traumatic brain injury increases galanin and galanin receptor mRNA (Kawa et al., 2016; Kawa et al., 2020), and galanin receptor mRNA increases after chronic mild stress (Wang et al., 2016). From a clinical perspective, this increase in galanin may be part of a larger response mechanism to rebalance galanin function to increase GalR2 activity and decrease Gal1R activity. For example, the commonly prescribed anti-depressant fluoxetine (a selective serotonin reuptake inhibitor) and electroconvulsive shock increases DRN galanin and GalR2 binding, whereas GalR2 knockout mice show enhanced depressive-like symptoms (Lu et al., 2005a; Lu et al., 2008). In tandem, DRN GalR1 expression is downregulated after stress, and its recruitment appears to account for increases in avoidance-like behaviors (Medel-Matus et al., 2017; Morais et al., 2016). In sum, the galanin system within the DRN has significant control over appetitive and aversive behaviors, with functional interactions between multiple receptor subtypes biasing behavioral responses toward one phenotype or the other. Future studies should clarify how these galanin systems converge in the DRN to impact behavior, as well as dissociate how incoming versus outgoing galanin systems regulate behavioral phenotypes.

### 3.4 Neuropeptide Y

#### 3.4.1 Expression patterns

Early studies established the presence of Neuropeptide Y (NPY) in the DRN across species (Figure 1), including rats (Smith et al., 1994; Yamazoe et al., 1985), lemurs (Bons et al., 1990), newts (Perroteau et al., 1988), and lampreys (Chiba, 1999). NPY in the DRN often colocalizes with serotonin (5-HT), suggesting functional interactions (Blessing et al., 1986). NPY exerts its effects in the brain mainly through the NPY receptor subtypes Y1, Y2, Y4, Y5, and Y6 (Díaz-Cabiale et al., 2011). Of these receptors, mRNA for Y1, Y2, and Y5 have been found throughout the DRN (Díaz-Cabiale et al., 2011; Gustafson et al., 1997; Kishi et al., 2005; Parker and Herzog, 1999). Detailed colocalization studies are lacking but Y2 receptor binding is enriched on glutamatergic neurons within the DRN, suggesting a possible relationship (Yoon et al., 2013).

#### 3.4.2 Functional effects

NPY exerts diverse effects in the DRN, with implications for stress, feeding, and social behaviors. Receptor-level studies reveal distinct roles for Y1 and Y2 receptors in the DRN. Functional studies suggest that NPY modulates GPCR but not amino acid signaling via Y2 in DRN neurons (Kombian and Colmers, 1992) with Y2 receptor activation suppressing food intake via glutamatergic neurons (Nectow et al., 2017). Y1 receptor activation leads to reduced maternal care in *ad libitum*-fed dams and suppressed male sexual behavior in fed mice (Inaba et al., 2016; Muroi and Ishii, 2015). Functional effects of Y5 receptors in the DRN have yet to be tested. Recent work found a unique population of NPY-expressing neurons in the DRN that are activated by stress and when they were exogenously activated through either chemogenetics or optogenetics they were able to improve stress resilience, including alleviation of stress-induced hypophagia, by interacting with Y2 receptors in the PVT and lateral hypothalamus (Zhang et al., 2024c). Additional work has found reduced DRN NPY levels in chronic pain and social fear conditioning models, further highlighting its critical role in DRN-mediated behavioral regulation (Hamann et al., 2022; Upadhya et al., 2009). Collectively, these data indicate that NPY within the DRN may act as an “aversion buffering” system. During endogenous recruitment, NPY may facilitate resilience-type phenotypes, whereas its loss (e.g., during chronic pain) allows for unusually high aversion. Future work is needed to elucidate the therapeutic potential of NPY within the DRN.

### 3.5 Additional neuropeptides

Although the DRN has been the focus of hundreds of studies, as this review illustrates, there is a significant paucity for what we know regarding DRN neuropeptides. Here, we briefly describe several poorly characterized DRN neuropeptides that are well known to impact reward and aversion in other brain circuits, including cholecystokinin, neurotensin, somatostatin, and Substance P.

#### 3.5.1 Cholecystokinin

Cholecystokinin (CCK) peptide was originally isolated from the gastrointestinal system, but is found throughout the central nervous system and densely in the DRN in rodents (Innis et al., 1979) and birds (Lovell and Mello, 2011). Functional CCK receptors reside within the DRN, and CCK application activates DRN 5-HT neurons primarily via CCK_*A*_ receptor (Boden et al., 1991). However, CCK itself does not colocalize with 5HT-containing neurons (van der Kooy et al., 1981). There is some variable colocalization of CCK with neurotensin and dopamine within the DRN (Seroogy et al., 1988). This expression pattern suggests that CCK-expressing neurons may be a distinct subpopulation within the DRN that could have unique effects on behavior. Limited behavioral studies have shown that DRN CCK has diverse behavioral effects. For example, CCK-positive neurons have been found to project from the arcuate nucleus to 5-HT cells within the DRN (Sim and Joseph, 1991) and systemic antagonism of CCK_*A*_ receptor potentiates intra-DRN 5-HT1A agonist-induced food intake (Currie and Coscina, 1995) and activation of DRN CCK+ neurons suppressed food intake (Chowdhury et al., 2025). However, direct administration of CCK to the DRN has no effect on food intake (Blevins et al., 2000), suggesting DRN CCK receptors may have divergent roles from the CCK peptide produced by DRN cell bodies and released downstream elsewhere. The mRNA for CCK is increased in the DRN following stress such as social isolation, chronic pain, or nocebo nausea (Del Bel and Guimarães, 1997; Keay et al., 2021; Zhang et al., 2024b). These DRN CCK-producing neurons have been found to project to the paraventricular thalamus, which is an important node for emotional and motivated behaviors (Bhatnagar et al., 2000; Otake, 2005). Altogether, while evidence indicates a complex role for CCK in DRN, more studies are needed to clarify its precise contributions and mechanisms of action.

#### 3.5.2 Neurotensin

Neurotensin expression in DRN was first described in the 1970s and 80s (Beitz, 1982; Jennes et al., 1982; Moyse et al., 1987; Sutin and Jacobowitz, 1988; Uhl et al., 1979b). Its receptors were mapped several decades later, with neurotensin receptor 2, but not neurotensin receptor 1, expressed in DRN (Boudin et al., 1996; Sarret et al., 2003). While its overall pharmacology is complex (Gereau et al., 2023), the net effect of neurotensin stimulation in DRN is to increase the excitability of serotonin neurons (Jolas and Aghajanian, 1996), particularly in the ventromedial portion of DRN. Functionally, neurotensin stimulation increases passive avoidance (Shugalev et al., 2005; Shugalev et al., 2007) and is decreased after adolescent ethanol intake (McClintick et al., 2015). However, intracerebroventricular neurotensin blunts stress evoked increases in serotonin biomarkers, leaving unresolved whether neurotensin primarily increases or decreases raphe activity.

#### 3.5.3 Somatostatin

Like neurotensin, descriptions of somatostatin expression in DRN of multiple species can be traced back to the 1980s (Araneda et al., 1999; Cooper et al., 1981; Cotter and Laemle, 1987; Fu et al., 2010; Smith et al., 1994; Spangler and Morley, 1987; Taber-Pierce et al., 1985; Weindl et al., 1984). While somatostatin likely has direct functional effects in DRN, to date very few studies have examined specific roles for the different receptor subtypes. This largely forces us to speculate about potential functions. For example, systemic administration of a somatostatin receptor 4 (SSTR4) agonist leads to an anxiolytic phenotype and increases c-fos in DRN (Scheich et al., 2016), perhaps indicating that somatostatin may promote serotonin activity. In cortex, hippocampus, and hypothalamus slices somatostatin induces the release of 5-HT (Tanaka and Tsujimoto, 1981), suggesting it would do the same in DRN cell bodies, but this remains to be tested. Similarly, ventrolateral PAG/lateral DRN infusions of SST reduces thermal nociception, again reducing aversive responses (Helmchen et al., 1995). In line with the concept that DRN somatostatin may reduce aversive processing, somatostatin, SSTR1, and SSTR2 gene expression is reduced after adolescent ethanol intake (McClintick et al., 2015). Excessive ethanol intake is highly correlated with increased pain and anxiety-like behaviors, but whether there is a causal role for somatostatin in DRN on these psychological phenotypes remains untested.

#### 3.5.4 Substance P

Substance P is a G_*q*_-coupled receptor that has broad expression throughout the brain. While the peptide is predominantly confined to the ventrolateral PAG, it does infiltrate the DRN (Figure 1) (Baker et al., 1991; Magoul et al., 1988; Smith et al., 1994). The neurokinin receptors (NK1, NK2, NK3) are also expressed in DRN, with NK1 being particularly enriched in DRN (Lacoste et al., 2006; Léger et al., 2002; Maeno et al., 1993). Curiously, both agonism and antagonism of NK1 receptors have been shown to increase serotonin neuron excitability (Conley et al., 2002; Haddjeri and Blier, 2001; Liu et al., 2002; Santarelli et al., 2001), perhaps suggesting that different receptors or cell types may dramatically shift ultimate physiology (Commons and Valentino, 2002; Valentino et al., 2003). Broadly, the Substance P system has been associated with various disease states, including depression and chronic pain (Adell, 2004). It is therefore somewhat surprising that comparatively little has been done to assess how Substance P/NK receptors functionally contribute to behavioral phenotypes. While general antagonism or knockout of NK1 receptors increases serotonin transmission and reduces aversive behavioral profiles, causally linking these phenomena have yet to be tested (Santarelli et al., 2001; Santarelli et al., 2002; Schank et al., 2015).

## 4 Conclusion

The dorsal raphe nucleus is an extraordinarily complex structure that has been implicated in mood, affect and motivation for decades. Though initially described in terms of its serotonergic architecture, further anatomical analyses now include the lateral wings and non-serotonergic neuropeptidergic circuits. Unfortunately, despite conscientious descriptions of dorsal raphe neuropeptide anatomy and convincing evidence that neuropeptides can profoundly shape behavioral phenotypes in other limbic brain circuits, comparatively little has been done to study dorsal raphe neuropeptides. By far the most extensive literature relates to the role of CRF, yet even these studies are predominantly framed from a serotonin-centric perspective. We have illustrated this point in Table 1, where we have summarized the currently known effects of each of the discussed neuropeptides in the DRN on either rewarding or aversive behaviors as well as their known effects on serotonin neuron excitability or release. It should be appreciated that all of the peptides have been at least studied in relation to their effect on serotonin, but not on their DRN-specific roles in behavior. This myopic approach could potentially limit our appreciation for how neuropeptides systems may contribute to behavioral phenotypes independent of, or perhaps in parallel with, serotonin.

**TABLE 1:** Summary of effects of DRN neuropeptides on rewarding or aversive behaviors and 5-HT signaling.

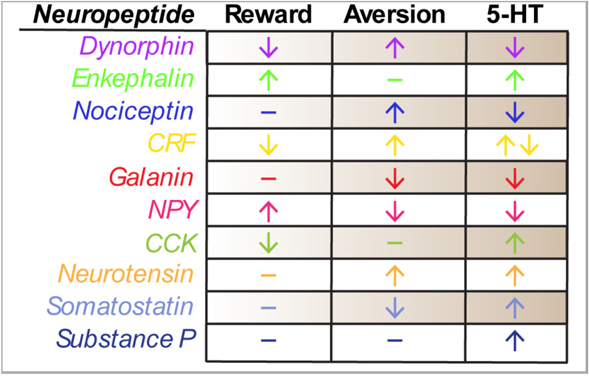

Symbols indicate that either peptide infusion or receptor activation in the DRN has been found to increase (↑) or decrease (↓) the corresponding behavior type or serotonin neuron excitability or release. Dashes (–) indicate that there is no published data on the peptide’s action on behavior or 5-HT flux in the DRN.

As experimental neuroscience technology continues to develop, it is imperative that we appreciate how neuropeptides are directly impacting physiology and behavior. Recent advances in biotechnology make it possible to study these neuropeptides with unprecedented specificity in behavioral models. The advent of CRISPR-Cas9 has allowed for the specific knockdown of precursor mRNA for peptides in combination with genetic Cre mouse lines. This technology provides the opportunity to investigate their role in behavioral paradigms and physiological functions in specific circuits or cell types. Previous interrogation of neuropeptidergic circuits was accomplished through pharmacology. Though receptor selective, the inability to target specific cell types pre- or post-synaptically has always limited interpretability. Fortunately, the recent development of DARTs (drugs acutely restricted by tethering) directly address this major confound. Specifically, DARTs use a bacterial enzyme to capture and tether drugs to defined cell surfaces, allowing researchers to pharmacologically target specific proteins on specific cells (Shields et al., 2017; Shields et al., 2024). As this technology continues to be optimized and used in more model systems, it will be interesting to see how it shapes our understanding of neuropeptide signaling. Anatomically, the last decade of in situ hybridization techniques have advanced to allow for much higher resolution and throughput of transcriptomic studies. Since many peptides can be difficult to measure (due to fast degradation, small molecular size, or natural biochemical instability), approaches targeting the mRNA have allowed for much more robust localization within neural circuits with single-cell resolution (DeLaney et al., 2018). Most recently the development of neuropeptide biosensors has invigorated neuropeptide research (Dong et al., 2024; Zhou et al., 2024; Wang et al., 2023). These fluorescent GPCR sensors allow for the detection of neuropeptide binding on a precise sub-second timescale with single cell resolution. Notably, a suite of sensors based on opioid receptors have been developed and validated: δLight, μLight, κLight (Dong et al., 2024) and NOPLight (Zhou et al., 2024). The implementation of these tools is only in its infancy, but they have already been used to confirm that dynorphin is released in the NAc shell in response to aversive stimuli such as a foot shock and revealed a shift in dynorphin dynamics throughout fear learning, wherein the κLight signal became stronger in response to the cue rather than the shock over more trials (Dong et al., 2024). This insight into the release dynamics of dynorphin during behavior can also be applied to the DRN to elucidate the contribution neuropeptides have to its roles in reward and aversion. These and other neuropeptide biosensors are poised to push the field forward and as they are optimized and implemented in various models, it will lead to a deeper understanding of neuropeptide dynamics throughout the brain (Dong et al., 2024; Siuda et al., 2015; Wang et al., 2023; Zhou et al., 2024).

Collectively, the dorsal raphe neuropeptides described in this review appear to converge on a similar functional role. Specifically, they primarily seem to bias responding, rather than generate phenotypes per se. For example, μ opioid or galanin stimulation attenuates aversive responses, whereas CRF promotes it in response to specific stimuli. Perhaps more importantly, the mechanisms through which each of these neuropeptides produces these effects are extraordinarily nuanced. They often include both pre- and post-synaptic mechanisms, as well as serotonergic and non-serotonergic targets. While some of these effects can be explained by biased activity on GABAergic cells or by the selective expression of one receptor versus another, there is much to do to within each neurochemical class to disentangle their respective mechanisms. Further complicating these analyses, some of these neuropeptides have a degree of promiscuity or cross-system interaction. In the case of opioids, individual precursor peptides can be cleaved into a myriad of products, leaving unresolved whether an “enkephalinergic” neuron is behaving as mu opioid receptor (i.e., met-enkephalin) or delta opioid receptor agonist (i.e., leu-enkephalin). Similarly, neurotensin has fairly selective receptors (NTSR1 and NTSR2), but is also known to act on sortilin, a receptor canonically shown to be involved in sorting proteins (Nykjaer and Willnow, 2012). CRF receptors can be activated by both CRF or urocortin, the latter of which has one isoform (urocortin 2) that preferentially binds to CRF2 over CRF1 (Kovács et al., 2025). Such complicated relationships between neuropeptides and their “target” receptors will likely define the future of neuropsychiatric drug development. As our molecular and signaling tools become more sophisticated, we may be able to develop positive or negative allosteric modulators that more efficiently target and bias receptor function without needing to oversaturate unnecessary subsystems. Looking forward, partnerships between medicinal chemists, behavioral neuroscientists, and molecular tool developers will be essential for continuing the advancement of neuropeptidergic research. Ultimately, these collaborations may reveal novel mechanisms or points of access for future therapeutic development and will positively shape the next several decades of neuropsychiatric research.
